# Adeno-Associated Virus (AAV-DJ)—Cryo-EM Structure at 1.56 Å Resolution

**DOI:** 10.3390/v12101194

**Published:** 2020-10-20

**Authors:** Qing Xie, Craig K. Yoshioka, Michael S. Chapman

**Affiliations:** 1 Department of Biochemistry & Molecular Biology, Oregon Health & Science University, Portland, OR 97239, USA; xieq@ohsu.edu; 2 Department of Biomedical Engineering, Oregon Health & Science University, Portland, OR 97239, USA; yoshiokc@ohsu.edu; 3 Department of Biochemistry, University of Missouri, Columbia, MO 65211, USA

**Keywords:** electron microscopy, refinement, gene therapy, vector

## Abstract

Adeno-associated virus is the leading viral vector for gene therapy. AAV-DJ is a recombinant variant developed for tropism to the liver. The AAV-DJ structure has been determined to 1.56 Å resolution through cryo-electron microscopy (cryo-EM). Only apoferritin is reported in preprints at 1.6 Å or higher resolution, and AAV-DJ nearly matches the highest resolutions ever attained through X-ray diffraction of virus crystals. However, cryo-EM has the advantage that most of the hydrogens are clear, improving the accuracy of atomic refinement, and removing ambiguity in hydrogen bond identification. Outside of secondary structures where hydrogen bonding was predictable a priori, the networks of hydrogen bonds coming from direct observation of hydrogens and acceptor atoms are quite different from those inferred even at 2.8 Å resolution. The implications for understanding viral assembly mean that cryo-EM will likely become the favored approach for high resolution structural virology.

## 1. Introduction

Interest in the structures of viruses goes back to the dawn of molecular biology and the hope for insights into the nature of living things from their crystallization [1]. By the early 1960s, the first atomic structures of proteins were revealed [2], and thoughts were turning to viruses. Symmetry became clear as nature’s key to the assembly of protective protein coats that could encapsidate virus-encoding genomes [3]. A young Michael Rossmann was harnessing symmetry as a means toward protein structure determination [4] that would, many years later, lead to the largest crystal structures, those of viruses. Faster progress towards understanding viral assembly, albeit at lower resolution, came through the development of electron microscopy (EM) approaches [5]. It would be another decade before technical developments by Rossmann and others led to the first virus high resolution crystal structures [6,7]. Both technologies have continued to develop and have been the foundations on which structural virology is built. Broadly, crystallography provides atomic detail where possible, and EM provides access to interesting structures that are too large or heterogenous. Their complementarity was illustrated in a joint study locating proteins within the adenoviral capsid [8], and recently, the numbers of viruses subject to both approaches has risen dramatically.

There are now approaching 1000 structures of viral assemblies (mostly icosahedral) in the Protein Data Bank [9], nearly 400 of which are X-ray crystallographic, and about 550 are by cryo-EM. At high resolution, there are three X-ray crystallographic structures beyond 1.9 Å: Coxsackievirus A24 at 1.4 Å [10], Satellite Tobacco Mosaic Virus [11] and Satellite Tobacco Necrosis Virus [12], both at 1.45 Å resolution. The EM structures are often at lower resolution, reflecting both past history of the technology, and its application to larger assemblies, those less symmetrical, and in complex with host molecules, all of which increase the challenges of crystallization and diffraction methods. The 2018 publication of a sub-2Å AAV2 structure indicated that EM technology was fast catching up [13]. The capabilities of the new cryo-EM technology [14] has been widely embraced, sometimes championed, by crystallographers, including those vested in crystallographic development, like Michael Rossmann [15]. Our own experimental analysis of the gene therapy vector, AAV-DJ got somewhat ahead of capabilities for the refinement of atomic structures against the highest resolution cryo-EM data. Here we report an analysis, now complete at 1.56 Å resolution. We describe the refinement process and what can be seen from cryo-EM at this near-atomic resolution.

AAV is a small single-stranded DNA parvovirus that attracted attention, because it infected humans without causing recognizable disease [16]. Three decades of development have culminated in the use of recombinant forms as vectors for the first FDA-approved in vivo gene therapies of genetic diseases, most notably in 2019 for a treatment of spinal muscular atrophy (SMA) [17,18]. AAV-DJ is a recombinant chimeric mix of natural serotypes 2, 8 and 9, selected from a randomized library for liver tropism and immune evasion [19]. Like the natural type-species, AAV-2, it attaches to extracellular heparanoids and became a model system in our laboratory for studying the glycan interactions [20,21,22]. Before the cryo-EM “resolution revolution” [23], studies of viral interactions with di- to penta-saccharides pushed the then-limits, and it was helpful to have the increased EM contrast and superior alignment of recombinantly expressed virus like particles (VLPs) over otherwise identical DNA-containing wild-type viruses.

Given its relevance to gene therapy, it is not surprising that the initial 3.2 Å crystal structure of AAV-2 [24] has been followed by 11 crystal structures of 6 natural serotype variants, some at resolutions of 2.6 Å [25,26]. There are 40 structures of AAV variants or complexes in the EM data bank, ranging in resolution mostly from 1.86 Å [13], then from 2.4 to 20 Å [27], three-quarters of which supported deposition of atomic coordinates. AAV is proving to be amenable to high resolution cryo-EM, even though heterogeneity would have suggested that it not be the best candidate. Alternate splicing and start codons result in three viral proteins in ~1:1:10 ratio of VP1:VP2:VP3, where VP1 and VP2 are N-terminally extended, the larger VP1 including a phospholipase A2 domain implicated on entry in endosomal escape [28]. While a fraction of VP2 N-termini could occupy pores on the 5-fold axes, the VP1-unique regions are thought to be usually sequestered inside the capsid until needed. Possible locations for VP1 and VP2-unique N-terminal regions have been proposed from diffuse structures in low resolution cryo-EM [29], but neither crystallography nor cryo-EM has shown any part of the VP1/2-unique regions definitively at high resolution. It is not known whether the minor capsid proteins occupy special locations in the otherwise icosahedrally-symmetric shell, and, by convention, residues for all subunits are numbered from the start of VP1, the first 200+ invisible.

The final step in structure determination, by crystallography or cryo-EM, is atomic refinement, in which the model is computationally adjusted for better fit to the experimental data while retaining excellent stereochemical geometry. In recent years, an adaptation of the crystallographic refinement package, Phenix, has become very popular in cryo-EM, and is noted for both excellent stereochemical restraints and speed [30]. The latter is achieved with target function that drives atoms along the gradient in the Coulombic potential map that is very fast, because it involves evaluations only at atom centers. For robust refinement of model disorder parameters and experimental resolution, the RSRef refinement method was more appropriate, with a real-space target function that compares potential calculated from the atomic model and electron scattering factors at all surrounding grid points in the map [31]. High resolution refinement with RSRef is currently implemented as an extension of the CNS suite [32]. Software updates were needed for then unchartered EM resolutions, for the inclusion of hydrogens, alternative rotamers and other features of high resolution structure.

## 2. Materials and Methods

### 2.1. Sample Preparation

Empty capsids of AAV-DJ were expressed in Sf9 insect cells, a baculovirus expression system, and purified using three rounds of CsCl density gradient ultracentrifugation, followed by heparin affinity chromatography, as previously described [27,33]. Capsid VLPs were dialyzed into 50 mM HEPES, 25 mM MgCl_2_, 25 mM NaCl, pH = 7.4. EM copper grids were R2/2 200 mesh from Quantifoil (Jena, Germany). AAV-DJ (3 μL at 0.6 mg/mL) was applied to copper grids that had been glow discharged in 75/25 percent Ar/O. Two aliquots were applied to the grid (to increase particle density) with manual blotting between. Grids were then vitrified in liquid nitrogen-cooled ethane using an FEI Vitrobot (FEI, Hillsboro, OR, USA) with a single 3 s blot of force 1 at 100% humidity.

### 2.2. Image Acquisition

Data were collected using a pixel size of 0.514 Å on a FEI Titan Krios (Thermo Fisher, Inc., Hillsboro, OR, USA) at 300 kV, using a Falcon 3 camera (Thermo Fisher, Inc.) with a total dose of ~30 e^−^/Å^2^ fractionated across 200 frames. The camera dose rate was ~0.5 e^−^/pixel/s. Defocus was randomly in the nominal range of 0.8 to 2.6 μm. Images were acquired in EPU (Thermo Fisher, Inc.) without the use of image shift. Coma-free alignment and objective astigmatism where corrected using Sherpa (Thermo Fisher, Inc.).

### 2.3. Image Processing

**Figure 1:**
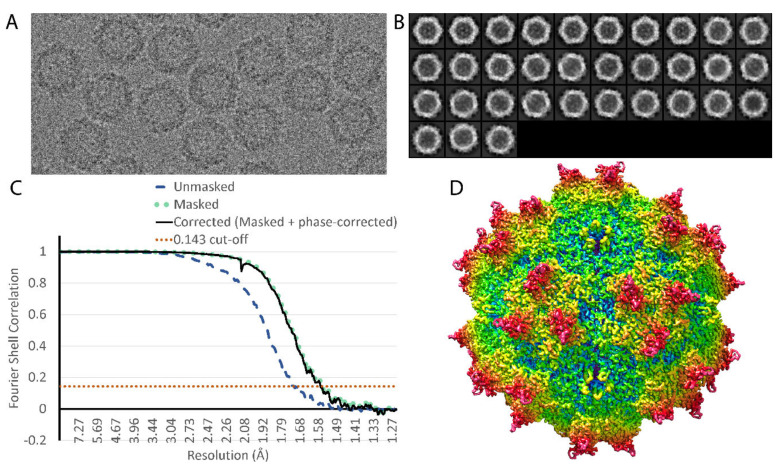
Cryo-EM reconstruction. (**A**) Motion-corrected image, integrated from frames; (**B**) 2D class averages calculated from 75,316 particles; (**C**) the gold standard FSC [47] gives an estimate of resolution at 1.56 Å; and (**D**) the reconstruction, rainbow colored according to distance from the center of the particle. The view is along a 2-fold symmetry axis. Spikes surrounding each 3-fold axis can be seen left and right of center, and 5-fold pores can be seen above and below center.

The AAV-DJ native dataset yielded 2241 movies. This initial dataset was refined to a resolution of ~2.2 Å before anisotropic magnification was observed to be preventing further progress. Distortion was estimated as ~0.9% and correction applied to the raw movies using magdistort binaries [34]. Processing was done within Relion 3.0 [35], with motion correction performed using Motioncor2 [36] and contrast transfer function (CTF) estimation done using Gctf [37]. DoGPicker [38] was used to pick ~70,000 potential particles and 4 rounds of 2D classification generated templates that were then used to re-pick 75,316 potential particles in Relion (Figure 1, Table 1). From here, multiple rounds of 2D classification and 3D classification with C1 symmetry were used to remove outliers, resulting in 48,209 particles after deduplication. These particles refined to ~2.2 Å with I1 symmetry, and subsequent refinements of beam tilt and per-particle CTF brought the resolution to 1.8 Å, further particle-polishing and subsequent re-refinement of CTF brought the resolution to 1.70 Å and a final reconstruction using Ewald’s sphere correction [39] ended at 1.56 Å. The map used for modeling was sharpened using the volume whitening routine in cisTEM [40].

### 2.4. Atomic Modeling and Refinement

Envelope corrections and resolution, together with the effective EM magnification (at the start), were least-squares refined periodically against the emerging atomic model, using RSRef [31]. Calibration of the magnification was against the prior 2.8 Å cryo-EM structure of a glycan complex [22], and ultimately back to an external crystallographic standard, the AAV2 crystal structure [24]. Resolution is refined by optimizing the parameters of a Butterworth low-pass filter applied to the atomic model, until its density optimally agrees with the experimental reconstruction.

Starting from the prior 2.8 Å structure of a glycan complex, the atomic model was refined by optimization of the agreement between model-calculated and the sharpened experimental Coulombic potential map, using the real-space RSRef method described in the Introduction [31]. The first batch used simulated-annealing torsion angle dynamics [32] with a slow cooling protocol starting at a temperature of 5000 K (assessed as better than 10,000 K). Coot [41] “manual” rebuilding was alternated with RSRef refinement, using the unsharpened map where the sharpened map was unclear. The second and subsequent batches of refinement used gradient descent optimization instead of simulated annealing. Prior to the third batch, solvent waters were modeled into unoccupied peaks >1.8 σ where there was the potential for hydrogen bonding. Bound ions and riding hydrogens [42] were also added. The latter necessitated the addition of hydrogen electron scattering factors to RSRef [43], and use of the “allhdg5-4” topology and parameter files for CNS force field stereochemical restraints [44]. Before the next iteration of RSRef, hydrogen positions were optimized using an Amber force field as implemented in Phenix geometry minimization [45], harmonically restraining all non-hydrogen atoms. The energy minimized histidine protonation states were assessed: five were confirmed in the map, four were ambiguous, and one needed reconfiguring, along with surrounding waters. These protonation states were fixed by declarations as “HISD” or “HISE” to CNS. All atoms were refined on subsequent RSRef optimization, but water molecules, whose hydrogens were ill-defined in the map, remained close to their Amber-optimized orientations.

Segments of five loops were disordered in the sharpened reconstruction, backbone falling below 1 σ: residues 217–222, 264–268, 327–333, 453–458, and 705–708, totaling 24 of 532 (4%) amino acids. Residues 217–220 had not been observed in prior AAV-DJ structures. At least backbone density was clear in the unsharpened reconstruction, so the final batch of refinement was divided into two, first refining these segments into the unsharpened reconstruction while all other atom positions were harmonically restrained strongly. Then the selections were inverted for refinement of all but the less-ordered segments to the sharpened reconstruction. Alternate side chain rotamers were rechecked, and nine were retained with user-estimated relative occupancies.

The above represents a conservative model parameterization, because, with cryo-EM, cross-validation is not available as a check on over-fitting. Individual atomic B-factors were approached similarly. In the absence of cross-validation, the restraint weight on the similarity of neighboring B-factors (0.018) was adjusted to yield a root mean square deviation (RMSD) less than the crystal structures of AAV-2 and AAV-3B (1.6 Å^2^) [24,26].

RSRef supports the refinement not only of individual atomic B-factors, but an imaging envelope correction (overall B-factor), and resolution, implemented as a 5th order Butterworth low-pass filter [31,46]. These are covariant and cannot all be refined together. For a model-based estimate of resolution, it was refined against the 249 atoms of the β-barrel at the core of the VP subunit, subject to a constraint that <B> ≥ 0.0. This embodies the premise that the best estimate of experimental resolution would come from the parts of structure least likely to be disordered. Indeed, the mean B-factor for these atoms is 3.3 Å^2^, close enough to 0.0 to indicate that map-sharpening had been near optimal and that a model-based resolution estimate would be a good approximation.

The observability of solvent molecules, hydrogens, et cetera, in the reconstruction was quantified in the following way. Model-map correlation coefficients were calculated as the occupancies of classes of atoms were varied: protein hydrogens, solvent hydrogens, solvent oxygens and (for comparison) protein backbone and protein side chain carbons. Correlation coefficients were estimated from the calculated Coulombic potential at all grid points in the experimental reconstruction within 2 Å of any atom, such that the same map volume was used for all calculations. Occupancies were varied between 1.0 and 0.0, normalizing the change in correlation coefficient by the total number of electrons in the atom set.

Accession numbers: An atomic model will be available from the protein data bank (http://www.rcsb.org) PDBid 7KFR. EM reconstructions will be available from the EMDataBank (http://www.ebi.ac.uk/pdbe/emdb/) with accession number EMD-22854.

## 3. Results

By FSC_0.143_ gold standard [47], the resolution is estimated to be 1.56 Å (Figure 1, Table 1). It is only recently with pre-print reports of apo-ferritin assemblies that this resolution has been exceeded for cryo-TEM of biomolecular assemblies [48,49]. There have been many measures of resolution in cryo-EM [50], and with the technological advances in recent years, there is growing appreciation of molecular features resolvable at the more common circa 3 Å resolution, we are in unchartered territory near 1.5 Å. The community has been shown how the Coulombic potential (EM maps) can differ from electron density (crystallography) [51]. Furthermore, EM resolution is conventionally measured in terms of signal-noise, in reciprocal space, and so it is an “average” between regions that may have substantial variation in local effective resolution [52]. A refined atomic structure allows a real-space evaluation with refinement of the parameters of a low pass filter applied to calculation of Coulombic potential from the atomic model for best agreement with the experimental reconstruction. An FSC_0.143_ calculated between two half data sets corresponds to the resolution where C_ref_, the cross-correlation between a full set and a noise-free reference, falls to 0.5 [53]. Although there is not strict equivalence, one might therefore expect correspondence between FSC_0.143_ and d_0.5_, the point at which a fitted 5th order Butterworth low pass filter half attenuates Fourier contributions to calculation of the Coulombic potential from the atomic coordinates [31]. When optimized using the full atomic model, d_0.5_ refines to 1.44 Å.

Local resolution is also a combination of multiple effects including (a) molecular disorder, (b) sample preparation, (c) microscope transfer/point spread functions as well as (d) alignment and other errors in computational reconstruction [54,55]. They can be difficult to tease apart, because of covariance of atomic displacement parameters (B-factors), envelope, or sharpening corrections and other resolution attenuation. It was postulated that these could be factored into molecular (a) and experimental (b–d) components if a significant part of the structure was highly ordered, as evidenced by mean refined B-factor approaching zero (without an envelope correction). The 249 atoms within the nine strands, βA–βG, of the core subunit jellyroll approximate this, with mean B, <B> = 3.3 Å^2^. The refined d_0.5_ is only marginally different at 1.43 Å, suggesting that on completion of refinement, the resolution estimate reflects the best experimental resolution, and that the molecular disorder is parameterized through variation in the atomic B-factors. Note that the model-referenced estimate (1.44 Å) differs somewhat from the FSC_0.143_, reflecting both differences in their means of calculation, and that the most ordered parts of the structure are seen with greater clarity than average.

**Figure 2:**
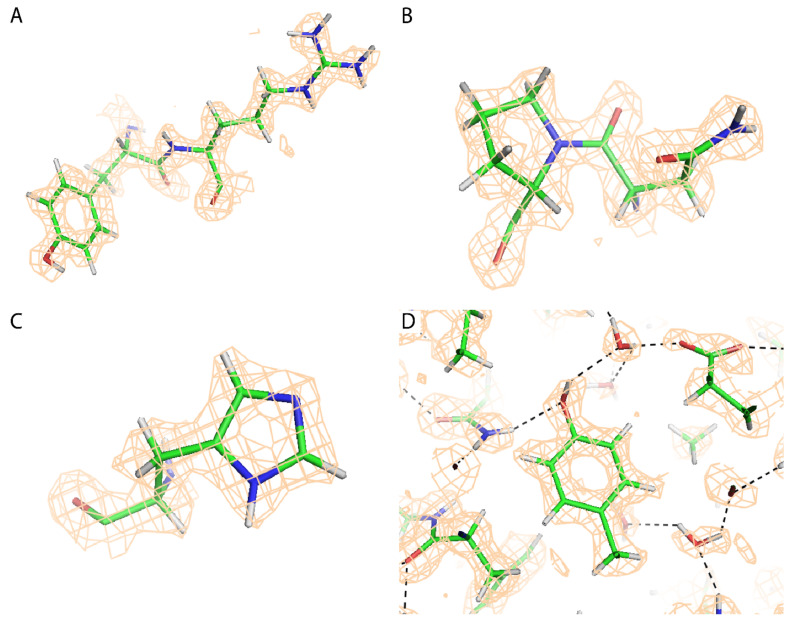
Fit of the atomic model to the map, contoured at 1.5 σ with atoms color-coded: carbon, green; nitrogen, blue; oxygen, red; and hydrogen; grey. (**A**) Tyr_485_-Arg_486_: bulges are apparent for all aliphatic and guanidinyl hydrogens and for the tyrosine hydroxyl; (**B**) Asn_520_-Pro_521_: the tetrahedral shape of the sp^3^ carbons define the pucker of this proline which is clearly in cis configuration; (**C**) His_360_: the map is suggestive that there is a hydrogen at N_δ_, and that the N_ε_ is predominantly deprotonated; (**D**) Tyr_283_: the aromatic hydrogens are clearly visible, as is the hydroxyl, oriented for hydrogen bond donation to a solvent water that bridges to the carboxylate of Asp_285_.

A priority was to establish what could be interpreted with confidence in EM reconstructions at this resolution. This started during the atomic refinement, because we needed to know how to appropriately parameterize the model. Inspection of the map clearly showed the presence of a first hydration shell of solvent water molecules bound to many of the polar protein groups (Figure 2D). In the absence of cross-validation methods, modeling was conservative, adding solvent molecules only where peaks in the map exceeded 1.8 σ, totaling 265 per protein subunit or an average of one water per two amino acids. Presumptive divalent ions could also be seen, coordinated by histidine ring nitrogens. Unexpected at this resolution was the prevalence of features corresponding to hydrogen atoms. In X-ray crystallography, 1.2 Å resolution is generally considered the starting point for modeling hydrogens, with complete structures expected only beyond 1 Å resolution. Indications of hydrogens had been reported in an EM structure at 1.86 Å resolution [13], but now, as we approach 1.5 Å resolution, hydrogen locations through most of the protein are clear to model (Figure 2D).

**Figure 3:**
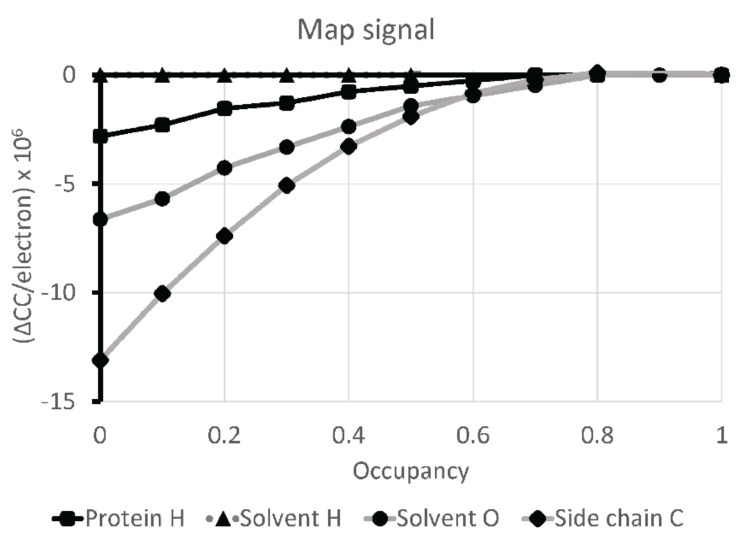
Signal for hydrogen atoms. As the group occupancy is changed from 1 (right) to 0 (left), the change in map-model cross-correlation coefficient (ΔCC) is plotted, normalized per electron in the atom group. For protein hydrogens, the map has signal ¼ of that expected relative to side chain carbons, while the oxygens of solvent molecules are at ½ the expected signal.

As resolution improves, one expects a gradual strengthening of features that would be impossible to see at lower resolution. The strengths of features per atom type are calibrated in Figure 3 to those of side-chain carbons, and we see that protein hydrogens are at about a quarter of full signal, solvent oxygens are at half, and that, on average, there is no signal for the more disordered solvent hydrogens. Their placement depends completely upon the Amber molecular mechanics calculation.

At the end of atomic refinement (Table 2), deviations from ideal geometry are very modest, both for attributes that are stereochemically restrained (bond lengths, angles, contacts) and those that are unrestrained and used as a read-out on model quality (Ramachandran φ, ψ and side chain rotamer outliers). For a reconstruction that is optimally sharpened so that it reveals the detailed features, high resolution noise is amplified, so cross-correlation coefficients (CC) are lowered. For the unsharpened map, the overall CC is 0.90 when d_0.5_ refined to 2.7 Å using map grid points within 1.8 Å of any atom. Against the sharpened map, the overall CC is 0.80 for grid points within 2.0 Å of atoms. However, the quality varies from excellent in the β-barrel (CC = 0.88 for grid points within 2 Å of the 249 atoms) to marginal, the marginal regions includingthe 24 disordered loop residues with breaks in backbone continuity that are only breached in the unsharpened map.

While the locations of hydrogens are predominantly clear, they are not as well enclosed within a map isocontour as other atoms. There are multiple causes. Hydrogen atoms have only a single electron/proton, so their Coulombic potential is weaker than non-hydrogen protein atoms, even if the experimentally observed signal were not one quarter of that to be expected (above). Furthermore, it has been noted by others that the orbitals of a σ-bonded hydrogen are not centered on the nucleus, but skewed towards the covalent bond [56,57]. This leads to an appearance in X-ray structures of hydrogens being 10% closer to their covalently bonded partner, and EM structures may see some of a similar effect. Some choose to display and restrain towards a shortened bond, but Figure 2 shows nucleus-defined atom centers.

Now with a very high resolution structure as a yardstick, it is enlightening to analyze the accuracy of prior AAV-DJ structures at 2.8 Å and 4.5 Å, [20,22], the former representing a resolution that is often a goal in the emerging post “resolution revolution” era of cryo-EM, while the latter represents the 5 Å average of structures deposited to EMDB in 2019–2020. It is sobering that the root mean square deviation (RMSD) between the 2.8 Å and 1.56 Å structures is as high as 0.9 Å, given estimates that stereochemical restraints in protein crystallography support accuracy 5–8-fold beyond the nominal experimental resolution [31,58,59,60]. Upon analysis, the AAV-DJ comparison is driven by 10% of the atoms that are outliers with an RMSD of 2.3 Å, while 90% have an RMSD of 0.35 Å (Table 3). If it is assumed that the deviations can be attributed to resolution-proportional and independent errors in the two structures, the estimated coordinate errors for the 90% are 0.17 Å and 0.31 Å, 9-fold better than the respective nominal 1.56 Å and 2.8 Å resolutions. Outliers in comparing crystal structures have been reported, but the fraction outside the expected distribution [58] is greater for our cryo-EM structures.

**Figure 4:**
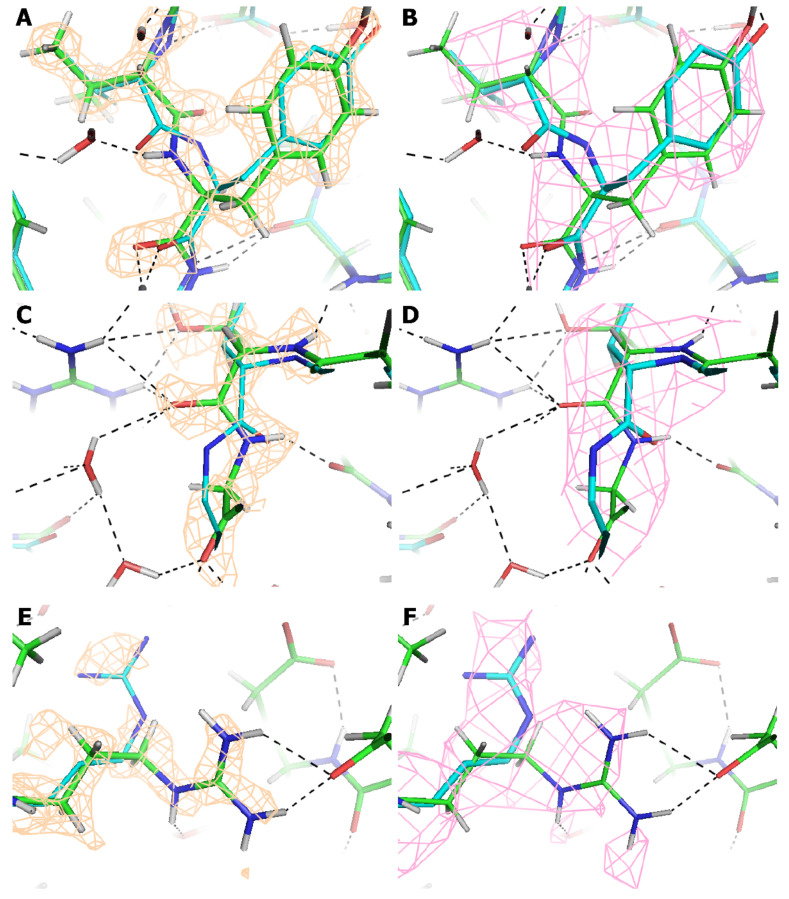
Improvements in the structure between 2.8 Å and 1.56 Å resolution. Paired images show the 1.56 Å (brown, left) and 2.8 Å (pink, right) reconstructions with the refined atomic models with green C_α_ (1.56 Å) or cyan (2.8 Å). (**A**,**B**) Peptide bond Val_613_-Tyr_614_, both maps contoured at 2.0 σ. (**C**,**D**) Peptide at the tight turn Thr_407_-Gly_408_, both maps contoured at 1.5 σ. (**E**,**F**) Side chain of Arg_391_, both maps contoured at 1.1 σ.

The largest outliers (5–10 Å) are in side-chains, usually where there is little indication in any of the maps as to which rotamer should be chosen. Figure 4E,F show a 5 Å change in an arginine side chain that is likely somewhat disordered, but where the higher resolution map gave clear indication of the need for a change. Evidence is not only in the map for the guanidinium, but also from the tetrahedral hydrogens that confirm the positions of C_β_ through C_δ_, consistent only with the 1.56 Å resolution structure. As for backbone outliers, about 30% are in less ordered parts of AAV-DJ, in the 5% of the structure that was refined against the unsharpened reconstruction, because it was clearer than the sharpened map. Other backbone outliers come from the many regions in the 2.8 Å map where the map has a tubular backbone without definition of carbonyl oxygens. In retrospect, we see that the peptide bonds were flipped in the 2.8 Å atomic model. Figure 4 illustrates two regions. Figure 4A clearly shows the correct peptide orientation. At 2.8 Å (Figure 4B) it would be difficult to distinguish correct from incorrect. Panels C and D show an example of where the 2.8 Å structure followed that of homologous AAVs, but it was not clear that a correction was needed until the 1.56 Å resolution reconstruction. There are many other peptide bonds where the 2.8 Å map gives no indication of carbonyl orientation, but where the change in the 1.56 Å resolution structure is more subtle than a peptide flip. Even through not optimally configured at 2.8 Å, it is only a few percent of NH-OC backbone-backbone hydrogen bonds that are not correctly identified at 2.8 Å, providing that generous angular cut-offs are used [61]. So, secondary structure is predominantly identified correctly at 2.8 Å. This feeds back to improve model quality, because, once recognized, even absent formal restraints, our a priori understanding of idealized secondary structure informs improved atom placement, mitigating ambiguity in the map.

**Figure 5:**
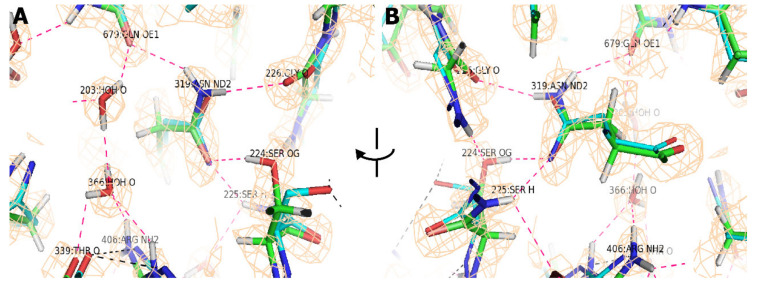
The hydrogen bond network near Asn_319_ defined at high resolution. Panels (**A**) and (**B**) are forward and reverse views of the same region, rotated about the vertical axis shown. The atomic models at 2.8 Å and 1.56 Å are shown with cyan and green carbons, respectively, overlaid on the 1.56 Å resolution map, contoured at 1.5 σ. The network of 12 hydrogen bonds implicated at 1.56 Å (pink dashes) is completely different from the 3 suggested at 2.8 Å (black dashes). There are three principle causes: (1) the water molecules bridging between Gln_679_ and Thr_339_/Arg_406_ of a neighboring subunit are apparent only at the higher resolution. Solvent hydrogen locations are not apparent in the map and were oriented by energy minimization. (2) At 2.8 Å, an incorrect rotamer was chosen for Asn_319_, with χ_2_ flipped by 180°. Even at 1.56 Å, the map gives little indication, but the changed rotamer has superior interactions with Gln_679_ and Ser_224_-Gly_226_. (3) There were two reasons that the latter were not recognized: (a) the O_γ_ of Ser_225_ was misplaced by 2 Å, with the correct rotamer only apparent at 1.56 Å; (b) The backbone for Ser_224_-Gly_226_ differs by 0.52 Å. In the 1.56 Å resolution map, both the carbonyl oxygen of Gly_226_ and the amino hydrogen of Ser_225_ are clear (see reverse view, panel (**B**)) in the map contoured at 1.5 σ, confirming the interpretation.

The improved resolution has greater impact upon the understanding of atomic interactions, where they are not clear in the lower resolution map, and there is not a priori information as to their nature. Figure 5 explains a typical situation where the hydrogen bonding network is completely different when seen at high resolution. At 2.8 Å the water molecules could not be modeled, and the rotamer of Asn_319_ had side chain dihedral angle, χ_2_ flipped, i.e., rotated by 180°. The correct asparagine rotamer is inferred from the interactions with Ser_224_-Gly_226_. This was not possible at 2.8 Å, because of backbone deviations of 0.52 Å (not particularly atypical with an RMSD of 0.42 Å). The advantage at 1.56 Å is clear definition of both the carbonyl oxygen of Gly_226_ and the amino hydrogen of Ser_225_ which precisely define the backbone location and settle the ambiguity at 2.8 Å resolution in rotamer choice and hydrogen bond interactions.

## 4. Discussion

Although the AAV-DJ structure represents a milestone, there is reason for optimism that it might prove, in due course, to lack exceptionality. While this is a robust viral capsid with the icosahedral symmetry that is highly advantageous in cryo-EM reconstruction, it is a relatively heterogenous virus with variant capsid proteins in unpredictable locations (see Introduction). Furthermore, the results were achieved with a microscope configuration that has become standard over the last couple of years. Approximately the same number of particles (47,000; 2.6 × 10^6^ asymmetric units) were used as in an earlier (2017) 2.8 Å structure determination [22], in other words, this is a standard data collection regime without extraordinary efforts to maximize the resolution. The results reported here have not depended on the very latest microscope developments that have supported apoferritin structures beyond 1.5 Å [48,49].

The structure contains many parts that reveal detailed features, such as hydrogen locations, with clarity that we would not have expected from X-ray structures at corresponding resolutions. However, comparisons of the different AAV-DJ structures show larger discrepancies than we might expect at 1.6–2.8 Å resolution. With AAV-DJ, about 10% of the reconstruction is disordered enough to affect atomic modeling. This appears to be a higher proportion than in crystal structures, and only about half become clear with high resolution EM data. It is not clear that biologist “consumers” of atomic structures will evaluate the experimental map directly, so it becomes a priority to develop annotations of the local confidence in EM-derived atomic models, so that detailed interpretations are not extended from robust regions into parts where conclusions might be fickle.

What biochemical insights come from observation of hydrogens? Recognizable tetrahedral shape removes ambiguity in modeling aliphatic groups (Figure 2A), and can provide unequivocal evidence for infrequent conformations such as cis-peptides (Figure 2B). In these cases, the positions of hydrogens are implicitly defined (completely) by the locations of neighboring heavy atoms. However, it is not just “riding” hydrogens that are observable. For much of the structure, those whose positions depend upon rotation about a next-nearest bond are also indicated. This can be significant for hydroxyl groups, where rotation about the hydroxyl bond determines which neighboring atoms could be hydrogen bond acceptors (or donors). Examples of tyrosine hydroxyl groups are shown in Figure 2A,D where the map indicates the direction of the hydrogen.

At this resolution of ~1.5 Å, we are also getting some indication of the presence, or not, of titratable protons. For seven of thirteen histidines in an AAV-DJ VP3, the map indicates which nitrogen(s) of a histidine side chain are protonated (Figure 2C); two others show the entire imidazole ring, but without distinctive hydrogens at N_ε_ or N_δ_; while four exhibit disorder that precludes imidazole proton assignment with confidence. None of the seven are seen in the fully protonated HisH^+^ form which is expected to be a minor fraction at the buffer pH 7.4, unless perturbed by the local environment. Two of the seven appear predominantly in the N3 tautomer (hydrogen at N_ε_) which is favored 4:1, absent perturbation, due to a slightly more basic pKa of the N3 tautomer [62], while five are the N1 tautomeric form (hydrogen at N_δ_), a ratio indicative of some perturbation. For six of the seven distinctive histidines, post facto, we see strong chemical rationale for the tautomer due to one or more of the following: (1) a glutamate or aspartate carboxylate oxygen within 3Å hydrogen-bonding distance of N_ε_ or N_δ_ and raising its pKa; (2) metal ion coordination by one nitrogen, lowering its pKa and raising the other [63,64]; (3) hydrogen bonding with a partner that is unambiguously a donor (amide nitrogen) or acceptor (carbonyl oxygen), implying, respectively, the absence or presence of a hydrogen at that histidine nitrogen; and (4) steric clash if protonated. One histidine is well solvated at both nitrogens, with no obvious perturbation, and it is in the favored N3 tautomeric form. There are no cases where map features are inconsistent with analysis of pK-perturbing interactions. Thus, while hydrogens are not clear for all histidines, all indications are that features seen are real and not false positives. Thus, we are observing, at 1.56 Å resolution, the effects of local environmental pKa perturbations of the nitrogen atoms of half of the histidines. This has exciting implications when high resolution studies of enzymes and channels become more routine, and one can imagine pH-dependent studies to analyze titratable protonation states.

The X-ray crystal structures of five different viruses/VLPs and their variants have been reported at better than 2 Å with three at slightly beyond 1.5 Å—Coxsackievirus A24v, Satellite Tobacco Mosaic Virus (STMV) and Satellite Tobacco Necrosis Virus (STNV) [10,11,12]. For none of these highest resolution virus structures has it been possible to visualize hydrogens or refine their atomic positions. A first indication of hydrogen atoms came with a 1.86 Å cryo-EM structure of an AAV variant [13]. Here, at 1.56 Å, the AAV-DJ structure is refined with a full complement of hydrogens, most of which are clearly defined in the map. This heralds a new era in virus structure with direct experimental observation leading to significantly improved characterization of the hydrogen bonding networks that are key to tertiary structure and assembly. This is coming from cryo-EM (rather than X-ray crystallography), because of the greater contribution of nuclear charge to the scattering of electrons compared to X-ray scattering which depends upon a single electron for a hydrogen atom [49,65,66]. At the forefront of EM resolution are structures of high symmetry, the 24-mer apoferritin and the 60-mer AAV, with distinct advantages in terms of protomer orientation distribution, alignment determination and potential for signal averaging. Their example is demonstrating that for other samples, instrumentation need not be limiting. Indeed the structure of a pentameric membrane protein has recently been reported at 1.7 Å resolution [48].

## 5. Conclusions

Even for those of us who came of age after development of protein crystallography, the progress in cryo-EM has been astonishing. Michael Rossmann and others of his generation took on the then seemingly impossible challenge of extending X-ray crystallography to mega-Dalton viral assemblies. It was their vision and success that became the foundation for structural virology. Notwithstanding his investment in crystallographic methodology, he was among the first to embrace complementary application of both approaches. As cryo-EM now becomes the choice even for high resolution virus structure, one suspects that he might have envisioned this before the rest of us, and that he would now be embracing the progress.

## Figures and Tables

**Table 1:** Cryo-EM data collection and processing.

Data Collection	
Magnification	155,000×
Voltage	300 kV
Electron exposure	30 e^−^/Å^2^
Defocus range	−0.8 to −2.6 μm
Pixel size	0.514 Å
(on refinement vs. atomic model:)	0.5105 Å
**Data Processing**	
Motion correction	Relion 3.0
Anisotropic magnification correction	
Distortion angle	5.3°
Percent distortion	0.91%
CTF estimation	
Resolution range	30 to 1.56 Å
Symmetry imposed	I1
Initial particle images	85,341
Final particle images	48,209
Map resolution	1.56 Å
FSC threshold	0.143

**Table 2:** Refinement of the atomic model.

Protein atoms/asymmetric unit (mean B-factor):	8144 (5.6 Å^2^)
Non hydrogen	4176 (5.3 Å^2^)
Core β-barrel	249 (3.3 Å^2^)
Water molecules	265 (11.9 Å^2^)
RMS bond length deviation from ideal	0.019 Å
RMS bond angle deviation from ideal	1.4°
Ramachandran outliers	1 (0.2%)
Side chains: multiple conformer/outliers	8/6 (1%)
Cross-correlation (model-map)	0.80
Resolution from model-map refinement (d_0.5_)	1.44 Å (core β-barrel)

**Table 3:** Comparison with lower resolution cryo-EM structures (RMS differences). For the 2.8 Å structure, both glycan-bound and unbound conformers were refined against a symmetrized partially bound reconstruction [22]. Compared here is the unbound conformer. Outliers are defined in the conventional statistical sense with differences >3rd quartile + 1.5 × (interquartile range).

Atoms	Cf. AAV-DJ at 4.5 Å (pdbID: 3J1Q) [20]	Cf. AAV-DJ at 2.8 Å resolution (pdbID: 5UF6) [22]
All	1.39 Å	0.87 Å
All, after rotamer inversion of (pseudo-) symmetrical side chains [58]	1.36 Å	0.80 Å
After outliers removed (% atoms)	1.06 (7%)	0.35 Å (10%)
ackbone	0.96 Å	0.42 Å
After outliers removed (% atoms)	0.87 (3%)	0.29 Å (6%)
C_α_	0.95 Å	0.35 Å
After outliers removed (% atoms)	0.86 (3%)	0.28 Å (5%)
